# Nociception and the neonatal brain

**DOI:** 10.1016/j.siny.2019.05.008

**Published:** 2019-08

**Authors:** Deniz Gursul, Caroline Hartley, Rebeccah Slater

**Affiliations:** Department of Paediatrics, University of Oxford, John Radcliffe Hospital, Headington, Oxford, OX3 9DU, United Kingdom

**Keywords:** Analgesia, Electroencephalography, Electromyography, Evoked potentials, Clinical trials, Development, Facial expressions, Magnetic resonance imaging, Near-infrared spectroscopy, Neonate, Neuroimaging, Nociception, Pain measurement

## Abstract

Measuring brain activity in infants provides an objective surrogate approach with which to infer pain perception following noxious events. Here we discuss different approaches which can be used to measure noxious-evoked brain activity, and discuss how these measures can be used to assess the analgesic efficacy of pharmacological and non-pharmacological interventions. We review factors that can modulate noxious-evoked brain activity, which may impact infant pain experience, including gestational age, sex, prior pain, stress, and illness.

## 1 Introduction

In adults, self-report of pain is the gold standard of pain reporting, and correlates strongly with pain-induced brain activity [1]. Given that the cortex is where nociceptive inputs are modulated and influenced to form subjective pain experiences [2], it follows that non-invasive brain imaging may provide the closest estimate of pain perception in the absence of a verbal report. This is of key utility in non-verbal neonates who cannot communicate their pain perception verbally, as alternative surrogate measures, such as facial grimacing, motor responses and autonomic activity, may not be as reliably linked to the perceptual experience, and may be more highly influenced by contextual factors [3].

Providing analgesia for infants is crucial, as pain in early life can cause negative short-term and long-term consequences. Short-term effects include decreased physiological stability, such as increased heart rate [4] and decreased respiration rate [5], and long-term effects include altered pain thresholds [[6], [7], [8], [9]], and neurocognitive development, including alterations in brain structure, behaviour, and cognitive ability [[10], [11], [12]]. These differences have been reported to be present in school-age children, and continued research is now beginning to report differences persisting into adulthood [13].

Infant pharmacokinetics and pharmacodynamics differ from those of adults [14], and providing analgesia to infants is not straightforward as it cannot be assumed that the analgesics that work in adults are efficacious or safe in infants [15]. Noxious-evoked brain activity is modulated by analgesics in both adults [[16], [17], [18], [19], [20]] and infants [21]. Therefore, brain imaging can potentially provide an objective and specific method of estimating infant pain experience and analgesic efficacy, and is of valuable utility to aid the discovery of effective analgesics [22]. Information on analgesic efficacy can then be used to determine whether the benefits of the intervention outweigh any potential adverse side effects [23].

In this review, we detail the different methods by which it is possible to measure pain-related brain activity in infants, and how these methods can be used to assess the efficacy of analgesics and non-pharmacological interventions. Additionally, we discuss the factors affecting the measurement of noxious-evoked brain activity and the assessment of infant pain.

## 2 Methods of pain assessment

### 2.1 Brain imaging

#### 2.1.1 NIRS (Near-infrared spectroscopy)

NIRS measures changes in cerebral oxygenation to infer functional brain activity, relying on the assumption that increased tissue oxygenation reflects an increase in blood flow due to increased neural activity. NIRS has been used in both adults [24] and infants to look at sensory evoked activity [25,26]. Due to its portability and ease of application, NIRS was the first measure of brain activity used to investigate whether afferent nociceptive input was transmitted to the infant cortex. This demonstration that noxious-evoked activity could be transmitted to the infant cortex – which is necessary for the experience of pain – was an important step forward in infant pain research. An optical sensor was placed on the scalp over the somatosensory cortex, which is located superficially and therefore more accessible to noninvasive optical techniques such as NIRS [27], and increased cerebral oxygenation was observed after clinically necessary noxious stimuli (heel lance blood tests and venipuncture), but not after non-noxious control stimuli [28,29].

NIRS has since been used to measure brain activity evoked by heel lances in independent samples of infants [30,31] and to other clinically necessary procedures, such as chest drain removal [32]. These studies have added further evidence to support the claim that the NIRS signals being measured reflect noxious-evoked haemodynamic changes in brain activity, and have demonstrated that pain-related NIRS signals can be observed in sedated infants despite dampened pain behaviours, highlighting that measures of brain activity may provide additional information about the infant pain experience. However, a limitation of NIRS is that the observed signals are based on the assumption that there is a direct correspondence between the increased blood flow and underlying brain activity, although this relationship may be influenced by many factors [33] and is more complex in the developing immature brain.

#### 2.1.2 EEG (electroencephalography)

EEG records the electrical activity of neural populations with an array of electrodes on the scalp and is also portable, but allows for a higher temporal resolution than NIRS. EEG has been used to record event-related potentials in both adults [20,34,35] and infants [36,37] following a range of stimuli, and EEG application is not associated with increased stress levels in infants [38].

In 2010, Slater et al. identified a noxious-evoked potential in infants, which occurs at ∼500 ms after a heel lance blood test over the vertex of the scalp [39] and is evident in single trials. This potential is also observed following low intensity experimental noxious stimulation, is graded with the intensity of the noxious stimulus [40], and is not observed following auditory, visual, or non-noxious tactile stimuli [21]. A similar pattern of noxious-evoked brain activity has also been recorded following vaccinations in infants aged 1 month to 1 year [41], demonstrating the commonality of this response following different noxious stimuli and in older age groups.

In order to use this evoked potential as an objective measure in clinical trials of analgesics it needs to be validated in independent samples of infants. A template of noxious-evoked brain activity [21] was derived from the brain activity of a sample of term infants following heel lance blood tests and experimental noxious stimuli, and then validated in four independent studies. Given a new EEG trace, this template can be projected onto the data and is essentially scaled to best fit the noxious-evoked response, providing a useful objective and automated method for quantifying the magnitude of infant noxious-evoked brain activity. In one validation study, the magnitude of the noxious-evoked brain activity quantified using the template was modulated by local anaesthetic, demonstrating its utility to objectively test analgesic efficacy. The reproducibility of this method has been demonstrated, with researchers from independent institutions recording noxious-evoked brain activity that can be reliably fitted with this template [42].

A limitation of this method is that measuring evoked activity at one location on the scalp does not reflect the full extent of nociceptive processing across the brain, and so analysis across a wider spatial area could improve our understanding and measurement of pain-related brain activity. Furthermore, the template can only be used to quantify noxious-evoked activity following acute, previously-characterised procedures. As many clinically relevant procedures occur instead over minutes or even hours, a different measure, such as changes in the EEG frequency, may be more suitable to characterise pain-related brain activity. Numerous studies have utilised EEG to characterise changes in the frequency domain of brain activity in adults, following both acute and sustained noxious stimulation (including intramuscular injections, thermal and laser stimulation), with observed changes in the alpha, beta, gamma, and delta bands [[43], [44], [45], [46], [47]]. Alpha power has been suggested to be predictive of subjective pain perception [48,49] and Misra et al. [50] utilised a machine learning approach to show that pain perception can be classified based on pain-related changes in the power of EEG bands, demonstrating that there are characterisable and specific pain-related changes in EEG power which could be used to create a signature to identify pain. An increase in gamma oscillations, consistent with the adult response, has been observed following heel lance in infants [51]. Further characterization of changes in the time-frequency domain in relation to different noxious stimuli may provide a surrogate marker of the ongoing pain experience in infants.

#### 2.1.3 fMRI (functional magnetic resonance imaging)

fMRI measures changes in blood oxygenation to infer changes in brain activity, commonly using a technique called blood oxygenation level dependent (BOLD) imaging. Changes in the BOLD signal can be localised with high spatial resolution, enabling the identification of anatomical brain areas that are active during particular states. In adults, brain regions that are active during reported pain have been identified, including the primary somatosensory cortex, prefrontal cortex, anterior cingulate cortex, thalamus, insula, and amygdala [2,52]. A neurological signature of pain, which can discriminate between pain and non-pain states, has been identified in adults and it has been suggested that such a signature would be useful in non-verbal populations [17,53,54]. The feasibility of scanning neonates in response to noxious stimulation has been established [55] and the methods for both acquisition and analysis have been optimised, including design of neonatal specific head coils [56], optimization of echo time [57], identification of the infant haemodynamic response function [58] and development of bespoke analysis pipelines [59]. The regions of the infant brain that are active following experimental noxious stimulation have been identified [[60], [61]] and closely resemble those active during the adult experience of pain, including both sensory and affective brain regions.

Development of an fMRI-based signature of pain-related brain activity in infants would provide an objective approach to investigating which aspects of pain-related brain activity are modulated by interventions. Adult studies have suggested that fMRI could be used to optimise central nervous system (CNS) drug development in early-stage clinical trials, by providing insight into how drugs affect the brain in order to identify and prioritize the development of more promising candidates [18,62,63]. In the future, application of such a method could be of use in infants, in whom an objective method of drug testing and drug development is even more critical due to their lack of ability to verbally communicate their pain perception.

### 2.2 Electromyography (EMG)

EMG involves the placement of electrodes on the skin overlying muscle to quantify muscle activity. In the field of pain, EMG is applied to limbs to measure reflexes, which occur in both adults and infants in response to noxious stimulation in order to protect the body by minimizing contact with potential harm. Infant reflexes exhibit intensity encoding, correlating with the intensity of the noxious stimulus eliciting the reflex [64]. Although the magnitude of the reflexes elicited by noxious stimuli correlate with noxious-evoked brain activity [40], reflexes, especially in younger gestational age infants, also occur in response to tactile stimulation [65], meaning that they are not as specific a reflection of noxious input as noxious-evoked brain activity.

### 2.3 Behavioural and physiological measures

Behavioural and physiological surrogate measures of infant pain are the most common measures used for clinical pain assessment. Factors such as facial expressions (including brow bulge, nasolabial furrow, eye squeeze, facial muscle tension, and grimace), limb and torso movements, cry, heart rate, and oxygen saturations are measured in order to estimate infant pain levels. There are many composite measures of pain, including the Premature Infant Pain Profile-Revised (PIPP-R) [66], Neonatal Infant Pain Score (NIPS) [67], Behavioural Indicators of Infant Pain (BIIP) [68], Echelle Douleur Inconfort Nouveau-Ne (EDIN) [69] and the COMFORT scale [70], and these take into account a range of behavioural and physiological variables to provide a better estimate of infant pain. However, neonatal staff report difficulty with assessing pain based on behavioural and physiological indicators [71], and behavioural and physiological measures may also reflect other emotions such as distress [72,73]. Investigating how these measures relate to noxious-evoked brain activity across different infant populations (including those with illness or cerebral pathology) may improve our understanding of these behavioural responses.

### 2.4 A multimodal approach to pain assessment

It is clear that pain elicits a range of responses across the infant central nervous system that can be reliably recorded. These different measures of pain-related brain activity are often concordant. For example, group-level measures of brain activity recorded by NIRS and EEG demonstrate correlation [31], and EEG-recorded noxious-evoked brain activity correlates with both reflex withdrawal [40] and facial expression changes [21,74]. However, this is not always the case – some individuals do not have correlated NIRS and EEG responses [31], noxious-evoked brain activity can be observed in the absence of facial expression responses[ [21,40,75]], behavioural and physiological measures are not always harmonious [76,77], and cry presence or amplitude does not correlate with noxious-evoked brain activity [78,79]. Additionally, contextual factors and interventions can disrupt the relationship between ordinarily concordant measures: stress [80] and prior pain [81] disturb the relationship between behavioural measures and noxious-evoked brain activity, sucrose reduces facial expression responses but does not alter noxious-evoked brain activity or reflex withdrawal [82], and gentle touch reduces noxious-evoked brain activity but does not appear to alter reflex withdrawal [83].

These disassociations could be due to a range of reasons, including low signal to noise ratios when necessarily considering single trial responses, the distinction between distress and pain, and the immaturity of the developing cortical and corticospinal connections [84,85]. The discrepancies between these measures highlight how pain-related activity at different sites across the body reflect different aspects of the pain experience. Furthermore, it demonstrates how these patterns of activity can be independently modulated by comfort measures, the environment, or prior experience. These observations emphasize the importance of considering a comprehensive multimodal approach to pain assessment to allow the best estimation of the infant pain. A multimodal approach to pain assessment is well suited to answering research questions and may be particularly important when considering analgesic drug trials. A carefully considered multimodal approach to analgesic assessment, including brain imaging as well as physiological and behavioural indicators, is most likely best able to detect potential adverse side effects of potential analgesics as well as assess efficacy [15].

## 3 Use of brain imaging to assess potential pharmacological and non-pharmacological interventions

Given the adverse short-term and long-term effects of pain [[4], [5], [6], [7], [8], [9], [10], [11], [12], [13]], and also the potential negative side effects of analgesics, more information on both drug efficacy and drug safety in infants is needed to balance analgesic efficacy with potential adverse drug effects [23]. Previous clinical trials using behavioural and physiological indicators of pain as study endpoints have not always provided clear answers regarding the efficacy of potential analgesics [22,[86], [87], [88]]. In this section, we discuss how the different methods of brain imaging could be used to better assess the efficacy of pharmacological and non-pharmacological methods of pain relief.

### 3.1 Sucrose

Oral sucrose and similar sugar solutions are commonly given to infants as a method of pain relief [89], and many studies demonstrate reduced pain-related behaviour and physiology associated with oral sucrose administration [90]. NIRS was the first method of brain imaging used to investigate the effect of sucrose on brain activity following heel lancing [91] with no significant difference reported in cerebral blood volume between the sucrose or placebo group, despite reduced heart rate and crying after sucrose administration. In agreement with this study, Beken et al. [92] investigated infant brain activity following venipuncture, and found that dextrose caused greater changes in cerebral blood volume in the left frontoparietal region of the brain, but did not alter cerebral blood flow or tissue oxygenation when compared with sterile water administration, despite dextrose-associated reduced behavioural scores. Due to its location, the increase in frontoparietal blood volume was interpreted as an effect of the sugar solution on the brain but not on pain processing, and it was concluded that dextrose did not alter pain-related brain activity.

However, Bembich et al. have also used NIRS to investigate the analgesic efficacy of glucose, as well as breastfeeding, in two studies suggesting that both do alter pain-related brain activity [93,94]. In the first study, oral glucose was found to block or weaken cortical activity following heel lancing, whereas breastfed infants showed widespread cortical activation but presented significantly less behavioural pain expressions. In the second study, the effect of maternal relationship was investigated, with infants being held during sucrose administration or breastfed (rather than fed expressed breastmilk) benefiting from the greatest analgesic effect.

Similarly, EEG recordings have also been used to investigate the effect of sucrose on noxious-evoked brain activity. Fernandez et al. [95] reported that sucrose attenuated a right frontal increase in EEG activation after heel lancing. In contrast, in 2010 Slater et al. [82] found that sucrose did not affect the magnitude of noxious-evoked brain activity, despite reducing pain-related behaviour concordant with previous investigations [90]. It is likely that the noxious-evoked brain activity quantified by Slater et al. provides a more specific measure of the nociceptive activity as compared with either the NIRS studies or the EEG spectral frequency changes considered by Fernandez and colleagues as the study by Slater and colleagues compared the evoked activity with a control stimulus and analysed only the noxious-specific component of the response. It seems likely that sucrose may reduce distress-related behaviour and physiology but not alter noxious-specific processing, which means it also may not protect against the long-term adverse consequences associated with early life pain and its effects on the brain [96]. Additionally, the benefits of sucrose use as a comfort method in infants needs to be carefully balanced with possible negative side effects in light of research suggesting that repeated sucrose administration leads to altered brain structure in animal models [97] and altered neurobehavioural development in 40 week old infants [98]. Further research remains to be done to determine the long-term effects of sucrose use in humans.

### 3.2 Topical local anaesthetic

The current literature, which has relied on behavioural and physiological measures of infant pain, is divided on the analgesic efficacy of topical local anaesthetic (LA) in infants, with some studies concluding that LA is effective for a range of acute needle-related procedures such as heel lances, cannulation, and venipuncture, and others concluding that it is not [87]. Noxious-evoked brain activity following experimental noxious stimuli is reduced by LA [21], demonstrating that LA is effective in blocking peripheral nociceptive input. The lack of observed pain relief in earlier studies could be due to factors such as the choice of LA, length of application time, the limited penetration depth relative to the noxious stimulus, or due to the non-specific behavioural and physiological measures used to assess analgesic efficacy: infants may cry and exhibit facial expressions and increased heart rate due to the general distress associated with clinical procedures (for example, from having their hand held firmly in position for cannulation), regardless of the level of pain they are perceiving [21,22]. However, further work is needed to investigate whether noxious-evoked brain activity following clinical stimuli, which have a deeper penetration and are higher intensity, is also reduced.

### 3.3 Morphine

As with local anaesthetic, the literature has not yielded conclusive results regarding the analgesic efficacy of morphine in infants [86], with some studies reporting that morphine does provide adequate analgesia [[99], [100], [101]], and others concluding that it is not an effective form of pain relief [102,103]. The Poppi (Procedural Pain in Premature Infants) trial, a blinded, randomised, placebo-controlled trial [15,104], aimed to investigate whether oral morphine was an efficacious and safe analgesic for procedural pain in non-ventilated premature infants and used a multimodal approach, including measuring brain activity, behaviour, physiology, and reflex withdrawal to assess the responses to heel lancing and Retinopathy of Prematurity (ROP) eye screening. The trial was stopped early due to profound respiratory side effects. Morphine did not alter the magnitude of noxious-evoked brain activity following heel lancing, nor did it alter the PIPP-R [66] behavioural scores to either heel lancing or ROP screening, though the trial was underpowered due to early cessation and it was therefore not possible to draw conclusions about the analgesic efficacy of morphine. Importantly this trial demonstrates the importance of taking a multimodal approach to analgesic assessment, including the thorough analysis of potential adverse effects and the utility of brain imaging. The trial concluded that oral morphine at a dose of 100 μg/kg should not be recommended for non-ventilated infants for ROP screening due to its risk of adverse effects.

### 3.4 Skin-to-skin contact

Skin-to-skin contact is a comfort measure which reduces behavioural and physiological measures of infant pain [105]. NIRS has been used to investigate the effect of skin-to-skin contract on brain activity in preterm neonates undergoing venipuncture [106]. A significantly smaller increase in oxygenated haemoglobin was found when the infants were being held by their mothers compared with when they were lying in their cot or incubator, consistent with a lower behavioural and physiology pain-related score. The authors concluded that skin-to-skin contact had a pain-relieving effect.

### 3.5 Slow touch targeted towards C-tactile fibres

Slow, gentle touch at a velocity of approximately 1–10 cm/s has been demonstrated to reduce both self-reports of pain and pain-related brain activity in adults [107,108]. This effect is believed to be mediated by C-tactile (CT) fibres, a subclass of mechanoreceptor in the skin which respond to the pleasant aspects of touch [109,110], encouraging affiliative behaviours [19]. In infants, gentle massage has been shown to reduce pain-related behaviours and physiology [[111], [112], [113], [114], [115]]. This may be related to activation of CT-fibres; noxious-evoked brain activity in term infants (in response to both experimental noxious stimulation and clinically required heel lance) is reduced by slow gentle brushing [83], performed at a rate known to activate CT-fibres in adults [110]. As pain relief interventions are limited in infants and non-pharmacological methods of pain relief do not hold the risk of adverse side effects, evaluating their efficacy is essential. Determining whether gentle touch also modulates noxious-evoked brain activity in preterm infants will be an important next step.

## 4 Factors affecting noxious-evoked brain activity and the measurement of infant pain

Many factors influence how an infant responds to noxious stimulation, including their gestational age, sex, prior pain exposure, illness, and stress levels. Here we describe how noxious-evoked brain activity may be influenced by some infant contextual factors and how this changes in relation to other pain-related responses.

### 4.1 Gestational age and sex

Noxious-evoked brain activity measured with both EEG and NIRS increases in magnitude and decreases in latency during preterm development [28,116]. Noxious-specific potentials measured with EEG are more likely to occur in older infants, maturing from the non-modality specific burst patterns of EEG, known as delta brushes, seen in infants below ∼34–37 weeks gestational age[ [116,117]]. In term infants, functional connectivity (measured with fMRI) between regions in the descending pain modulatory system (DPMS) has been associated with lower noxious-evoked brain activity, suggesting that the DPMS has an inhibitory influence on noxious responses from a young age and that the development of the DPMS during early infancy will influence the magnitude of observed noxious-evoked brain activity [61].

Reflexes also mature during the preterm period, decreasing in magnitude, duration and latency, increasing in activation threshold, and beginning to occur more discriminately after solely noxious events rather than also tactile stimulation [64,65,116]. This refinement is concomitant with an increase in the magnitude of noxious-evoked brain activity, suggesting that top-down modulation of reflexes may begin to emerge in early postnatal life, consistent with animal studies[ [84,85,116]]. However, a dissociation between noxious-evoked brain activity and reflex withdrawal has been observed following the application of CT-optimal touch in term infants [83], which reduced brain activity but not reflex withdrawal. This may be due to immature descending corticospinal tracts limiting communication between the brain and spinal cord [84,85] and suggests that top-down inhibition is not fully mature at term age.

Facial expressions similarly change with age: the latency to facial expression change decreases with postmenstrual age [118], the duration of brow bulge, eye squeeze, and nasolabial furrow in the 30 s post stimulus increases with age, and infants are more likely to exhibit a discriminate facial response (to noxious stimuli but not non-noxious touch stimuli) over ∼ 33 weeks gestational age, concomitant with the maturation of noxious-specific brain activity [74]. Some behavioural and physiological measures of infant pain, such as the PIPP-R [66] and EDIN6 scale [119], account for gestational age in their points systems, allowing for the fact that younger infants are less likely to respond.

Sex-dependent differences in brain activity responses to noxious stimuli have been observed using EEG [120], with female infants more likely to exhibit widespread rather than localised pain-related potentials. Several behavioural studies have also reported sex-dependent differences, with female infants displaying more pain-related facial features [121] and higher pitch crying [122] than male infants. In concordance with this, the adult literature also reports differences in male and female pain perception, and the intensity and spatial distribution of pain responses [123].

### 4.2 Stress, illness and prior pain

Infants who are under stress or sick display differing responses to noxious stimuli. Jones et al. [80] found that stress (measured utilising salivary cortisol samples) leads to a dissociation between noxious-evoked brain activity and noxious-evoked behaviour, measures of pain which are usually correlated, with an increase in noxious-evoked brain activity but not behaviour. This is consistent with adult evidence that stress enhances pain sensitivity [125], but could lead to inaccurate estimations of infant pain based on behavioural indicators - further demonstrating the importance of a multimodal approach to infant pain measurement.

Illness also affects measurements of pain, with extremely sick infants less able to mount facial or behavioural responses [27] and displaying altered cry acoustics [126]. Additionally, behavioural measures can be difficult to observe in instances where clinical equipment such as intubation is present and masking facial features or the ability to cry [127]. Future research is required to investigate noxious-evoked brain activity in these infants, shedding light on their pain experience.

Regarding prior pain, Slater et al. [6] used EEG to determine that noxious-evoked potentials following clinically necessary heel lances were larger in ex-premature infants at term-corrected age (having spent time in neonatal units undergoing painful procedures) than in age-matched term-born infants. Ozawa et al. [81] demonstrated that prior pain disrupts the relationship between cortical and behavioural measures of pain, indicating that prior experiences should be taken into account when assessing neonatal pain. Prior pain also influences behavioural pain scores in both premature infants [128] and term-born infants [129]. Moreover, these effects may continue into childhood and adulthood: children who have experienced early life pain display increased cerebral responses to pain [130] and pain catastrophising [131], and early life surgery is related to altered somatosensory processing in young adults [13].

## 5 Conclusion

Measures of noxious-evoked brain activity are a useful tool for infant pain assessment as they are objective and quantifiable, and changes in brain activity are likely to be related to the experience of pain. These features mean that brain activity is also well suited for investigating analgesic efficacy. Noxious-evoked brain activity is generally correlated with other indicators of infant pain, however, brain activity can be seen in the absence of these indicators, and is influenced by contextual factors such as infant age, sex, prior pain, stress levels, and illness. A multimodal approach to pain assessment, including the measurement of noxious-evoked brain activity, can provide the most comprehensive estimate of the infant pain experience.

## Conflicts of interest

The authors have no conflicts of interest. Deniz Gursul receives funding from St Cross College, University of Oxford. Caroline Hartley is funded by the Wellcome Trust and Royal Society. Rebeccah Slater is funded by the Wellcome Trust.

**Practice points**

• Neonatal staff should be aware that noxious-evoked changes in brain activity can be recorded in infants even when there are no visible pain-related behaviours.
• Neonatal staff should be aware that interventions (such as oral sucrose) may dampen pain-related behaviours without affecting noxious-evoked brain activity.
• Neonatal staff should be aware that contextual factors such as age, sex, prior pain, stress, and illness will influence how an infant responds to noxious input, and that these factors can disrupt the relationship between different measures of noxious-evoked activity.

**Research directions**

• Noxious-evoked changes in brain activity should be characterised following longer clinical procedures and post-operative pain.
• Different modalities of brain imaging should be developed to provide well-defined templates of noxious-evoked brain activity.
• Brain imaging should be included with other pain assessment measures in clinical trials that aim to assess the efficacy of analgesic interventions.

## References

[1] Coghill R.C., McHaffie J.G., Yen Y.-F. (2003). Neural correlates of interindividual differences in the subjective experience of pain. Proc Natl Acad Sci.

[2] Tracey I., Mantyh P.W. (2007). The cerebral signature for pain perception and its modulation. Neuron.

[3] Sellam G., Cignaccol E.L., Craigl K.D., Engbergl S. (2011). Contextual factors influencing pain response to heelstick procedures in preterm infants: what do we know? A systematic review. Eur J Pain.

[4] Waxman J.A., Pillai Riddell R.R., Tablon P., Schmidt L.A., Pinhasov A. (2016). Development of cardiovascular indices of acute pain responding in infants: a systematic review. Pain Res Manag.

[5] Craig K.D., Whitfield M.F., Grunau R.V., Linton J., Hadjistavropoulos H.D. (1993). Pain in the preterm neonate: behavioural and physiological indices. Pain.

[6] Slater R., Fabrizi L., Worley A., Meek J., Boyd S., Fitzgerald M. (2010). Premature infants display increased noxious-evoked neuronal activity in the brain compared to healthy age-matched term-born infants. Neuroimage.

[7] Maitre N.L., Key A.P., Chorna O.D., Slaughter J.C., Matusz P.J., Wallace M.T. (2017). The dual nature of early-life experience on somatosensory processing in the human infant brain. Curr Biol.

[8] Hermann C., Hohmeister J., Demirakça S., Zohsel K., Flor H. (2006). Long-term alteration of pain sensitivity in school-aged children with early pain experiences. Pain.

[9] Valeri B.O., Ranger M., Chau C.M.Y., Cepeda I.L., Synnes A., Linhares M.B.M. (2016). Neonatal invasive procedures predict pain intensity at school age in children born very preterm. Clin J Pain.

[10] Vinall J., Miller S.P., Bjornson B.H., Fitzpatrick K.P.V., Poskitt K.J., Brant R. (2014). Invasive procedures in preterm children: brain and cognitive development at school age. Pediatrics.

[11] Doesburg S.M., Chau C.M., Cheung T.P.L., Moiseev A., Ribary U., Herdman A.T. (2013). Neonatal pain-related stress, functional cortical activity and visual-perceptual abilities in school-age children born at extremely low gestational age. Pain.

[12] Grunau R.E., Whitfield M.F., Petrie-Thomas J., Synnes A.R., Cepeda I.L., Keidar A. (2009). Neonatal pain, parenting stress and interaction, in relation to cognitive and motor development at 8 and 18 months in preterm infants. Pain.

[13] Walker S.M., Melbourne A., O'Reilly H., Beckmann J., Eaton-Rosen Z., Ourselin S. (2018). Somatosensory function and pain in extremely preterm young adults from the UK EPICure cohort: sex-dependent differences and impact of neonatal surgery. Br J Anaesth.

[14] Kearns G.L., Abdel-Rahman S.M., Alander S.W., Blowey D.L., Leeder J.S., Kauffman R.E. (2003). Developmental pharmacology — drug disposition, action, and therapy in infants and children. N Engl J Med.

[15] Hartley C., Moultrie F., Hoskin A., Green G., Monk V., Bell J. (2018). Analgesic efficacy and safety of morphine in the Procedural Pain in Premature Infants (Poppi) study: randomised placebo-controlled trial. Lancet.

[16] Atlas L.Y., Whittington R.A., Lindquist M.A., Wielgosz J., Sonty N., Wager T.D. (2012). Dissociable influences of opiates and expectations on pain. J Neurosci.

[17] Wager T.D., Atlas L.Y., Lindquist M.A., Roy M., Woo C.-W., Kross E. (2013). An fMRI-based neurologic signature of physical pain. N Engl J Med.

[18] Duff E.P., Vennart W., Wise R.G., Howard M.A., Harris R.E., Lee M. (2015). Learning to identify CNS drug action and efficacy using multistudy fMRI data. Sci Transl Med.

[19] McGlone F., Wessberg J., Olausson H., Abraira V.E., Ginty D.D., Ackerley R. (2014). Discriminative and affective touch: sensing and feeling. Neuron.

[20] Truini A., Panuccio G., Galeotti F., Maluccio M.R., Sartucci F., Avoli M. (2010). Laser-evoked potentials as a tool for assessing the efficacy of antinociceptive drugs. Eur J Pain.

[21] Hartley C., Duff E.P., Green G., Mellado G.S., Worley A., Rogers R. (2017). Nociceptive brain activity as a measure of analgesic efficacy in infants. Sci Transl Med.

[22] Moultrie F., Slater R., Hartley C. (2017). Improving the treatment of infant pain. Curr Opin Support Palliat Care.

[23] Moultrie F., Shriver A., Hartley C., Wilkinson D., Ewer A.K., Rogers R. (2019 Feb). A universal right to pain relief: balancing the risks in a vulnerable patient population. Lancet Child Adolesc Heal.

[24] Ferrari M., Quaresima V. (2012). A brief review on the history of human functional near-infrared spectroscopy (fNIRS) development and fields of application. Neuroimage.

[25] Kusaka T., Kawada K., Okubo K., Nagano K., Namba M., Okada H. (2004). Noninvasive optical imaging in the visual cortex in young infants. Hum Brain Mapp.

[26] Sakatani K., Chen S., Lichty W., Zuo H., Wang Y.P. (1999). Cerebral blood oxygenation changes induced by auditory stimulation in newborn infants measured by near infrared spectroscopy. Early Hum Dev.

[27] Ranger M., Johnston C.C., Limperopoulos C., Rennick J.E., du Plessis A.J. (2011). Cerebral near-infrared spectroscopy as a measure of nociceptive evoked activity in critically ill infants. Pain Res Manag.

[28] Slater R., Cantarella A., Gallella S., Worley A., Boyd S., Meek J. (2006). Cortical pain responses in human infants. J Neurosci.

[29] Bartocci M., Bergqvist L.L., Lagercrantz H., Anand K.J.S. (2006). Pain activates cortical areas in the preterm newborn brain. Pain.

[30] Bembich S., Brovedani P., Cont G., Travan L., Grassi V., Demarini S. (2015). Pain activates a defined area of the somatosensory and motor cortex in newborn infants. Acta Paediatr.

[31] Verriotis M., Fabrizi L., Lee A., Cooper R.J., Fitzgerald M., Meek J. (2016). Mapping cortical responses to somatosensory stimuli in human infants with simultaneous near-infrared spectroscopy and event-related potential recording. ENeuro.

[32] Ranger M., Synnes A.R., Vinall J., Grunau R.E. (2014). Internalizing behaviours in school-age children born very preterm are predicted by neonatal pain and morphine exposure. Eur J Pain.

[33] Roche-Labarbe N., Wallois F., Ponchel E., Kongolo G., Grebe R. (2007). Coupled oxygenation oscillation measured by NIRS and intermittent cerebral activation on EEG in premature infants. Neuroimage.

[34] Patel S.H., Azzam P.N. (2005). Characterization of N200 and P300: selected studies of the event-related potential. Int J Med Sci.

[35] Bromm B., Scharein E. (1982). Principal component analysis of pain-related cerebral potentials to mechanical and electrical stimulation in man. Electroencephalogr Clin Neurophysiol.

[36] Shibasaki H., Miyazaki M. (1992). Event-related potential studies in infants and children. J Clin Neurophysiol.

[37] Tokioka A.B., Pearce J.W., Crowell D.H. (1995). Endogenous event-related potentials in term and preterm infants. J Clin Neurophysiol.

[38] Whitehead K., Jones L., Laudiano Dray P., Meek J., Fabrizi L. (2018). Full 10-20 EEG application in hospitalised neonates is not associated with an increase in stress hormone levels. Clin Neurophysiol Pract.

[39] Slater R., Worley A., Fabrizi L., Roberts S., Meek J., Boyd S. (2010). Evoked potentials generated by noxious stimulation in the human infant brain. Eur J Pain.

[40] Hartley C., Goksan S., Poorun R., Brotherhood K., Mellado G.S., Moultrie F. (2015). The relationship between nociceptive brain activity, spinal reflex withdrawal and behaviour in newborn infants. Sci Rep.

[41] Verriotis M., Fabrizi L., Lee A., Ledwidge S., Meek J., Fitzgerald M. (2015). Cortical activity evoked by inoculation needle prick in infants up to one-year old. Pain.

[42] Klarer N., Rickenbacher H., Kasser S., Depoorter A., Wellmann S. (2017). Electrophysiological measurement of noxious-evoked brain activity in neonates using a flat-tip probe coupled to electroencephalography. J Vis Exp.

[43] Zhang Z.G., Hu L., Hung Y.S., Mouraux A., Iannetti G.D. (2012). Gamma-band oscillations in the primary somatosensory cortex--A direct and obligatory correlate of subjective pain intensity. J Neurosci.

[44] Huishi Zhang C., Sohrabpour A., Lu Y., He B. (2016). Spectral and spatial changes of brain rhythmic activity in response to the sustained thermal pain stimulation. Hum Brain Mapp.

[45] Peng W., Hu L., Zhang Z., Hu Y. (2014). Changes of spontaneous oscillatory activity to tonic heat pain. PLoS One.

[46] Michail G., Dresel C., Witkovský V., Stankewitz A., Schulz E. (2016). Neuronal oscillations in various frequency bands differ between pain and touch. Front Hum Neurosci.

[47] Ploner M., Sorg C., Gross J. (2017). Brain rhythms of pain. Trends Cognit Sci.

[48] Nir R.-R., Sinai A., Moont R., Harari E., Yarnitsky D. (2012). Tonic pain and continuous EEG: prediction of subjective pain perception by alpha-1 power during stimulation and at rest. Clin Neurophysiol.

[49] Furman A.J., Meeker T.J., Rietschel J.C., Yoo S., Muthulingam J., Prokhorenko M. (2018). Cerebral peak alpha frequency predicts individual differences in pain sensitivity. Neuroimage.

[50] Misra G., Wang W., Archer D.B., Roy A., Coombes S.A. (2017). Automated classification of pain perception using high-density electroencephalography data. J Neurophysiol.

[51] Fabrizi L., Verriotis M., Williams G., Lee A., Meek J., Olhede S. (2016). Encoding of mechanical nociception differs in the adult and infant brain. Sci Rep.

[52] Apkarian A.V., Bushnell M.C., Treede R.-D., Zubieta J.-K. (2005). Human brain mechanisms of pain perception and regulation in health and disease. Eur J Pain.

[53] Brown J.E., Chatterjee N., Younger J., Mackey S. (2011). Towards a physiology-based measure of pain: patterns of human brain activity distinguish painful from non-painful thermal stimulation. PLoS One.

[54] Reddan M.C., Wager T.D. (2018). Modeling pain using fMRI: from regions to biomarkers. Neurosci Bull.

[55] Williams G., Fabrizi L., Meek J., Jackson D., Tracey I., Robertson N. (2015). Functional magnetic resonance imaging can be used to explore tactile and nociceptive processing in the infant brain. Acta Paediatr.

[56] Seghier M.L., Lazeyras F., Huppi P.S. (2006). Functional MRI of the newborn. Semin Fetal Neonatal Med.

[57] Goksan S., Hartley C., Hurley S.A., Winkler A.M., Duff E.P., Jenkinson M. (2017). Optimal echo time for functional MRI of the infant brain identified in response to noxious stimulation. Magn Reson Med.

[58] Arichi T., Fagiolo G., Varela M., Melendez-Calderon A., Allievi A., Merchant N. (2012). Development of BOLD signal hemodynamic responses in the human brain. Neuroimage.

[59] Baxter L., Fitzgibbon S., Moultrie F., Goksan S., Jenkinson M., Smith S. (2018). Optimising neonatal fMRI data analysis: design and validation of an extended dHCP preprocessing pipeline to characterise noxious-evoked brain activity in infants. Neuroimage.

[60] Goksan S., Hartley C., Emery F., Cockrill N., Poorun R., Moultrie F. (2015). fMRI reveals neural activity overlap between adult and infant pain. Elife.

[61] Goksan S., Baxter L., Moultrie F., Duff E., Hathway G., Hartley C. (2018). The influence of the descending pain modulatory system on infant pain-related brain activity. Elife.

[62] Borsook D., Becerra L., Hargreaves R. (2006). A role for fMRI in optimizing CNS drug development. Nat Rev Drug Discov.

[63] Carmichael O., Schwarz A.J., Chatham C.H., Scott D., Turner J.A., Upadhyay J. (2018). The role of fMRI in drug development. Drug Discov Today.

[64] Andrews K., Fitzgerald M. (1999). Cutaneous flexion reflex in human neonates: a quantitative study of threshold and stimulus-response characteristics after single and repeated stimuli. Dev Med Child Neurol.

[65] Cornelissen L., Fabrizi L., Patten D., Worley A., Meek J., Boyd S. (2013). Postnatal temporal, spatial and modality tuning of nociceptive cutaneous flexion reflexes in human infants. PLoS One.

[66] Stevens B.J., Gibbins S., Yamada J., Dionne K., Lee G., Johnston C. (2014). The premature infant pain profile-revised (PIPP-r). Clin J Pain.

[67] Lawrence J., Alcock D., McGrath P., Kay J., MacMurray S.B., Dulberg C. (1993). The development of a tool to assess neonatal pain. Neonatal Netw.

[68] Holsti L., Grunau R.E. (2007). Initial validation of the behavioral indicators of infant pain (BIIP). Pain.

[69] Debillon T., Zupan V., Ravault N., Magny J.F., Dehan M. (2001). Development and initial validation of the EDIN scale, a new tool for assessing prolonged pain in preterm infants. Arch Dis Child Fetal Neonatal Ed.

[70] Ambuel B., Hamlett K.W., Marx C.M., Blumer J.L. (1992). Assessing distress in pediatric intensive care environments: the COMFORT scale. J Pediatr Psychol.

[71] Boyle E.M., Bradshaw J., Blake K.I. (2018). Persistent pain in neonates: challenges in assessment without the aid of a clinical tool. Acta Paediatr.

[72] Gaspardo C.M., Chimello J.T., Cugler T.S., Martinez F.E., Linhares M.B.M. (2008). Pain and tactile stimuli during arterial puncture in preterm neonates. Pain.

[73] Ahola Kohut S., Pillai Riddell R. (2009). Does the neonatal facial coding system differentiate between infants experiencing pain-related and non-pain-related distress?. J Pain.

[74] Green G., Hartley C., Hoskin A., Duff E., Shriver A., Wilkinson D. (2018). Behavioural discrimination of noxious stimuli in infants is dependent on brain maturation. Pain.

[75] Slater R., Cantarella A., Franck L., Meek J., Fitzgerald M. (2008). How well do clinical pain assessment tools reflect pain in infants?. PLoS Med.

[76] Barr R.G. (1998). Reflections on measuring pain in infants: dissociation in responsive systems and “honest signalling”. Arch Dis Child Fetal Neonatal Ed.

[77] Morison S.J., Grunau R.E., Oberlander T.F., Whitfield M.F. (2001). Relations between behavioral and cardiac autonomic reactivity to acute pain in preterm neonates. Clin J Pain.

[78] Maitre N.L., Stark A.R., McCoy Menser C.C., Chorna O.D., France D.J., Key A.F. (2017). Cry presence and amplitude do not reflect cortical processing of painful stimuli in newborns with distinct responses to touch or cold. Arch Dis Child - Fetal Neonatal Ed.

[79] Relland L.M., Gehred A., Maitre N.L. (2018). Behavioral and physiological signs for pain assessment in preterm and term neonates during a nociception-specific response: a systematic review. Pediatr Neurol.

[80] Jones L., Fabrizi L., Laudiano-Dray M., Whitehead K., Meek J., Verriotis M. (2017). Nociceptive cortical activity is dissociated from nociceptive behavior in newborn human infants under stress. Curr Biol.

[81] Ozawa M., Kanda K., Hirata M., Kusakawa I., Suzuki C. (2011). Influence of repeated painful procedures on prefrontal cortical pain responses in newborns. Acta Paediatr.

[82] Slater R., Cornelissen L., Fabrizi L., Patten D., Yoxen J., Worley A. (2010). Oral sucrose as an analgesic drug for procedural pain in newborn infants: a randomised controlled trial. Lancet.

[83] Gursul D., Goksan S., Hartley C., Mellado G.S., Moultrie F., Hoskin A. (2018 Dec 17). Stroking modulates noxious-evoked brain activity in human infants. Curr Biol.

[84] Devonshire I.M., Kwok C.H.T., Suvik A., Haywood A.R., Cooper A.H., Hathway G.J. (2015). A quantification of the relationship between neuronal responses in the rat rostral ventromedial medulla and noxious stimulation-evoked withdrawal reflexes. Eur J Neurosci.

[85] Hathway G.J., Koch S., Low L., Fitzgerald M. (2009). The changing balance of brainstem-spinal cord modulation of pain processing over the first weeks of rat postnatal life. J Physiol.

[86] Bellù R., de Waal K.A., Zanini R. (2008). Opioids for neonates receiving mechanical ventilation. Cochrane Database Syst Rev.

[87] Foster J.P., Taylor C., Spence K. (2017). Topical anaesthesia for needle-related pain in newborn infants. Cochrane Database Syst Rev.

[88] Baarslag M.A., Allegaert K., Van Den Anker J.N., Knibbe C.A.J., Van Dijk M., Simons S.H.P. (2017). Paracetamol and morphine for infant and neonatal pain; still a long way to go?. Expert Rev Clin Pharmacol.

[89] Committee on Fetus and Newborn and Section on Anesthesiology and Pain Medicine C on NP (2016). Prevention and management of procedural pain in the neonate: an update. Pediatrics.

[90] Stevens B., Yamada J., Ohlsson A., Haliburton S., Shorkey A. (2016). Sucrose for analgesia in newborn infants undergoing painful procedures. Cochrane Database Syst Rev.

[91] Bucher H.-U., Von Siebenthal K., Keel M., W M., Duc G. (1995). Sucrose reduces pain reaction to heel lancing in preterm infants: a placebo-controlled. Randomized Masked Study.

[92] Beken S., Hirfanoğlu İ.M., Gücüyener K., Ergenekon E., Turan Ö., Ünal S. (2014). Cerebral hemodynamic changes and pain perception during venipuncture. J Child Neurol.

[93] Bembich S., Davanzo R., Brovedani P., Clarici A., Massaccesi S., Demarini S. (2013). Functional neuroimaging of breastfeeding analgesia by multichannel near-infrared spectroscopy. Neonatology.

[94] Bembich S., Cont G., Causin E., Paviotti G., Marzari P., Demarini S. (2018). Infant analgesia with a combination of breast milk, glucose, or maternal holding. Pediatrics.

[95] Fernandez M., Blass E.M., Hernandez-Reif M., Field T., Diego M., Sanders C. (2003). Sucrose attenuates a negative electroencephalographic response to an aversive stimulus for newborns. J Dev Behav Pediatr.

[96] Schneider J., Duerden E.G., Guo T., Ng K., Hagmann P., Graz M.B. (2018). PROCEDURAL PAIN AND ORAL GLUCOSE IN PRETERM NEONATES. Pain.

[97] Tremblay S., Ranger M., Chau C.M.Y., Ellegood J., Lerch J.P., Holsti L. (2017). Repeated exposure to sucrose for procedural pain in mouse pups leads to long-term widespread brain alterations. Pain.

[98] Johnston C.C., Filion F., Snider L., Majnemer A., Limperopoulos C., Walker C.-D. (2002). Routine sucrose analgesia during the first week of life in neonates younger than 31 weeks' postconceptional age. Pediatrics.

[99] Anand K.J., Barton B.A., McIntosh N., Lagercrantz H., Pelausa E., Young T.E. (1999). Analgesia and sedation in preterm neonates who require ventilatory support: results from the NOPAIN trial. Neonatal Outcome and Prolonged Analgesia in Neonates. Arch Pediatr Adolesc Med.

[100] Anand K.J.S., Hall R.W., Desai N., Shephard B., Bergqvist L.L., Young T.E. (2004). Effects of morphine analgesia in ventilated preterm neonates: primary outcomes from the NEOPAIN randomised trial. Lancet.

[101] Taddio A., Lee C., Yip A., Parvez B., McNamara P.J., Shah V. (2006). Intravenous morphine and topical tetracaine for treatment of pain in [corrected] neonates undergoing central line placement. J Am Med Assoc.

[102] Simons S.H.P., van Dijk M., van Lingen R.A., Roofthooft D., Duivenvoorden H.J., Jongeneel N. (2003). Routine morphine infusion in preterm newborns who received ventilatory support. J Am Med Assoc.

[103] Carbajal R., Lenclen R., Jugie M., Paupe A., Barton B.A., Anand K.J.S. (2005). Morphine does not provide adequate analgesia for acute procedural pain among preterm neonates. Pediatrics.

[104] Slater R., Hartley C., Moultrie F., Adams E., Juszczak E., Rogers R. (2016). A blinded randomised placebo-controlled trial investigating the efficacy of morphine analgesia for procedural pain in infants: trial protocol. Wellcome Open Res.

[105] Johnston C., Campbell-Yeo M., Disher T., Benoit B., Fernandes A., Streiner D. (2017). Skin-to-skin care for procedural pain in neonates. Cochrane Database Syst Rev.

[106] Olsson E., Ahlsén G., Eriksson M. (2016). Skin-to-skin contact reduces near-infrared spectroscopy pain responses in premature infants during blood sampling. Acta Paediatr.

[107] Liljencrantz J., Strigo I., Ellingsen D.M., Krämer H.H., Lundblad L.C., Nagi S.S. (2017). Slow brushing reduces heat pain in humans. Eur J Pain (United Kingdom).

[108] Habig K., Schänzer A., Schirner W., Lautenschläger G., Dassinger B., Olausson H. (2017). Low threshold unmyelinated mechanoafferents can modulate pain. BMC Neurol.

[109] Vallbo A., Olausson H., Wessberg J., Norrsell U. (1993). A system of unmyelinated afferents for innocuous mechanoreception in the human skin. Brain Res.

[110] Löken L.S., Wessberg J., Morrison I., McGlone F., Olausson H. (2009). Coding of pleasant touch by unmyelinated afferents in humans. Nat Neurosci.

[111] Jain S., Kumar P., McMillan D.D. (2006). Prior leg massage decreases pain responses to heel stick in preterm babies. J Paediatr Child Health.

[112] Diego M.A., Field T., Hernandez-Reif M. (2009). Procedural pain heart rate responses in massaged preterm infants. Infant Behav Dev.

[113] Hathaway E.E., Luberto C.M., Bogenschutz L.H., Geiss S., Wasson R.S., Cotton S. (2015). Integrative care therapies and physiological and pain-related outcomes in hospitalized infants. Glob Adv Health Med.

[114] Zargham-Boroujeni A., Elsagh A., Mohammadizadeh M. (2017). The effects of massage and breastfeeding on response to venipuncture pain among hospitalized neonates. Iran J Nurs Midwifery Res.

[115] Chik Y.-M., Ip W.-Y., Choi K.-C. (2017). The effect of upper limb massage on infants' venipuncture pain. Pain Manag Nurs.

[116] Hartley C., Moultrie F., Gursul D., Hoskin A., Adams E., Rogers R. (2016). Changing balance of spinal cord excitability and nociceptive brain activity in early human development. Curr Biol.

[117] Fabrizi L., Slater R., Worley A., Meek J., Boyd S., Olhede S. (2011). A shift in sensory processing that enables the developing human brain to discriminate touch from pain. Curr Biol.

[118] Slater R., Cantarella A., Yoxen J., Patten D., Potts H., Meek J. (2009). Latency to facial expression change following noxious stimulation in infants is dependent on postmenstrual age. Pain.

[119] Raffaeli G., Cristofori G., Befani B., De Carli A., Cavallaro G., Fumagalli M. (2017). EDIN scale implemented by gestational age for pain assessment in preterms: a prospective study. BioMed Res Int.

[120] Verriotis M., Jones L., Whitehead K., Laudiano-Dray M., Panayotidis I., Patel H. (2018). The distribution of pain activity across the human neonatal brain is sex dependent. Neuroimage.

[121] Guinsburg R., Peres C. de A., Branco de Almeida M.F., Xavier Balda R. de C., Berenguel R.C., Tonelotto J. (2000). Differences in pain expression between male and female newborn infants. Pain.

[122] Fuller B.F. (2002). Infant gender differences regarding acute established pain. Clin Nurs Res.

[123] Fillingim R.B., King C.D., Ribeiro-Dasilva M.C., Rahim-Williams B., Riley J.L. (2009). Sex, gender, and pain: a review of recent clinical and experimental findings. J Pain.

[125] Crettaz B., Marziniak M., Willeke P., Young P., Hellhammer D., Stumpf A. (2013). Stress-induced allodynia--evidence of increased pain sensitivity in healthy humans and patients with chronic pain after experimentally induced psychosocial stress. PLoS One.

[126] Stevens B.J., Johnston C.C., Horton L. (1994). Factors that influence the behavioral pain responses of premature infants. Pain.

[127] Ramelet A.-S., Abu-Saad H.H., Rees N., McDonald S. (2004). The challenges of pain measurement in critically ill young children: a comprehensive review. Aust Crit Care.

[128] Johnston C.C., Stevens B.J. (1996). Experience in a neonatal intensive care unit affects pain response. Pediatrics.

[129] Taddio A., Shah V., Gilbert-MacLeod C., Katz J. (2002). Conditioning and hyperalgesia in newborns exposed to repeated heel lances. J Am Med Assoc.

[130] Hohmeister J., Kroll A., Wollgarten-Hadamek I., Zohsel K., Demirakça S., Flor H. (2010). Cerebral processing of pain in school-aged children with neonatal nociceptive input: an exploratory fMRI study. Pain.

[131] Hohmeister J., Demirakça S., Zohsel K., Flor H., Hermann C. (2009). Responses to pain in school-aged children with experience in a neonatal intensive care unit: cognitive aspects and maternal influences. Eur J Pain.

